# Effectiveness of Family Coping Interventions in Improving Problem-Solving Skills in the Care of Children and Adolescent Cancer Survivors during and after Treatment: A Scoping Review

**DOI:** 10.3390/nursrep14030161

**Published:** 2024-08-28

**Authors:** Pedro Emílio Gomes Prates, Antonio Jorge Silva Correa-Júnior, Tatiana Mara da Silva Russo, Camila Maria Silva Paraizo-Horvath, André Aparecido da Silva Teles, Helena Megumi Sonobe

**Affiliations:** 1 School of Nursing of Ribeirão Preto (EERP), Collaborating Center of the Development of Nursing Research (PAHO-WHO), University of São Paulo (USP), São Paulo 14040-902, Brazil; antoniocorreajunior@usp.br (A.J.S.C.-J.); tatiana.russo@usp.br (T.M.d.S.R.); camilaparaizo@usp.br (C.M.S.P.-H.); andreteles@usp.br (A.A.d.S.T.); megumi@eerp.usp.br (H.M.S.); 2 Brazilian Society of Clinical Oncology (SBOC), São Paulo 01311-300, Brazil; 3 Laboratory of High-Throughput Functional Biology and Biobank (FUNDHERP), Ribeirão Preto Hemocenter Foundation, São Paulo 14051-140, Brazil

**Keywords:** survivors of childhood cancer, coping skills, problem-solving, adaptive behavior, family adaptation, review

## Abstract

(1) Context: Cancer triggers significant changes in family dynamics. It is noteworthy that coping and problem-solving skills, particularly in situations involving cancer in children and adolescents, have not been adequately explored in the context of family adaptation. This study aims to analyze the effectiveness of family interventions in coping to improve problem-solving skills in parents and/or caregivers of children and adolescents during and after oncological treatment. (2) Methods: This is a scoping review following the recommendations of the Joanna Briggs Institute and the Systematic Reviews and Meta-Analyses Extension for Scoping Review (PRISMA-ScR), from 2014 to 2024, in the databases LILACS, CINAHL, SCOPUS, Web of Science, and PUBMED. (3) Results: Forty-five studies were eligible. Coping strategies were categorized as follows: (1) positive attitudes (including a sense of courage and hope, family support to enhance resilience, and future planning), (2) caregiver empowerment (involving acceptance of diagnosis, emotional distancing, and coping through religiosity), and (3) communication skills (encompassing professional communication, horizontal dialogue with healthcare teams, and sincere communication with friends and family). (4) Conclusions: Over time, families develop coping and problem-solving strategies that influence changes in family functioning patterns, aiding them in accepting, reinterpreting, and reframing ideas and feelings associated with neoplasia.

## 1. Introduction

Pediatric cancer encompasses a wide range of malignant neoplasms with distinct characteristics, including varied incidence, diverse origins, different treatment options, variable overall survival rates, and potential acute and late adverse effects resulting from therapeutic interventions [1,2]. These neoplastic entities share a common propensity for unregulated proliferation of atypical cells and can manifest in any region of the body in individuals during childhood or adolescence. It is noteworthy that the demographic group studied, as defined by the guidelines of the Brazilian Society of Pediatrics, comprises individuals aged 0 to 19 years [3,4].

Although childhood and adolescent cancers often receive less attention due to their lower incidence compared to prevalent types in adult and elderly populations [5], there has been a significant improvement in the overall survival trajectory of these patients over the last five decades. In 2021, the overall survival rate reached 80%, contrasting with about 30% in the 1960s. However, despite this progress, childhood and adolescent cancer remain the second leading cause of death in children aged 5 to 14 years, both globally and in Latin America and the Caribbean [6].

In the context of providing care to patients with specific therapeutic needs, it is crucial to consider the demands of this group and the challenges faced by their caregiving families. These demands require constant and comprehensive attention, often resulting in physical and emotional burden for caregivers [7,8]. This burden can manifest through symptoms such as fatigue, stress, anxiety, depression, and social isolation, negatively impacting not only the caregivers’ quality of life but also that of the patients under their care. Furthermore, burden can be understood as a condition in which family members struggle to adequately meet the diverse needs of the patient [9].

Research dedicated to analyzing resilience and adaptation in the context of chronic conditions has revealed that, despite significant challenges, families have the capacity to develop behaviors and mobilize resources that alter their dynamics, making situations more manageable and acceptable [9,10,11]. This phenomenon can contribute to strengthening both parents and the family unit. In this context, it is crucial for professionals to be attentive to circumstances that may hinder or impede adaptation, encouraging families to adopt strategies that allow them to effectively cope with stressful situations [12,13,14].

Efforts directed at both individual and familial levels can be undertaken to mitigate the demands associated with childhood and adolescent cancer and to seek resources to deal with these situations. This approach is aligned with the Resilience, Stress, Adjustment, and Family Adaptation Model, which views such efforts as coping strategies [15]. Family coping entails coordinating behaviors aimed at problem-solving, with complementary individual efforts from its members, potentially resulting in a balance between demands and resources, and consequently, a reduction in faced difficulties [16,17]. Thus, coping refers to the way individuals interact with stressors and challenges, triggering various mechanisms to identify resources that help them develop enduring skills to manage crisis situations [18,19].

Thus, coping aims to explain the potential of parents and family members to deal with crisis situations, promoting understanding of the factors related to family adjustment and adaptation [15]. Coping and problem-solving (PSC) are understood in this research as actions for change. Their function is to restore and contribute to the balance between demands and resources while simultaneously removing or reducing the difficulties involved in the stressful situation [15].

Furthermore, it is crucial to emphasize that the manifestation of cancer within the family nucleus causes significant changes in family dynamics, necessitating a reorganization involving the redefinition of roles and responsibilities. This restructuring is necessary to empower the family to provide support to the affected individual, ensuring the satisfaction of their basic human needs such as hygiene, nutrition, rest, as well as the provision of emotional and financial support. Consequently, the daily demands associated with patient care impose a considerable burden of responsibility on the caregiver, often leading them to neglect their own needs in favor of the patient’s. Such a situation frequently results in physical, social, psychological, and spiritual overload for the caregiver [10,14].

Furthermore, it is important to emphasize that the dimensions of coping and problem-solving in contexts related to the diagnosis of childhood cancer [20,21,22] have not yet been adequately explored within the scope of family adaptation. This gap highlights the importance of conducting the present research, aiming to fill this knowledge gap and provide valuable insights for a more comprehensive understanding of the challenges faced by families in this specific context.

Furthermore, it is worth noting that this investigation is based on evidence regarding a set of actions, efforts, and behaviors established by families, which make situations related to childhood and adolescent cancer more manageable. Identifying, understanding, and anticipating the resources that families can use to adapt to the conditions associated with this disease will significantly contribute to guiding nurses in providing care and making decisions related to managing these situations.

This scoping review aims to analyze the effectiveness of family interventions in coping to improve problem-solving skills in parents and/or caregivers of children and adolescents during and after oncological treatment.

## 2. Materials and Methods

### 2.1. Ethical Aspects

As this is a scoping review, which used publicly available data and did not involve human subjects, there was no need for approval from the Research Ethics Committee (CEP). However, it is important to note that the studies selected for the final sample were duly referenced [23].

### 2.2. Study Design

This is a scoping review, developed following the recommendations of the international guideline Preferred Reporting Items for Systematic Reviews and Meta-Analyses Extension for Scoping Reviews (PRISMA-ScR) [24] and the Joanna Briggs Institute (JBI), 2020 version Reviewers Manual [25,26], with a research protocol registered on the Open Science Framework (OSF) platform [27]. It is noteworthy that a scoping review represents a novel approach to systematic literature reviews, with a growth in both national and international publications over the last decade. The method allows for examining evidence, existing gaps, and key concepts surrounding a specific study object [25,26]. During the construction of this study, the following structuring of steps was opted for, as conceptualized by Arksey and O’Malley: (1) establishment of the research question; (2) identification of relevant studies in national and international literature; (3) analysis and extraction of studies; (4) data organization; (5) compilation, synthesis, interpretation, and reporting of results [28].

### 2.3. Methodological Procedure

It is worth noting that, initially, a search was conducted in the scientific literature to identify reviews with a similar scope of research. Platforms such as the International Prospective Register of Systematic Reviews (PROSPERO), Open Science Framework (OSF), The Cochrane Library, JBI Clinical Online Network of Evidence for Care and Therapeutics (COnNECT+), and Database of Abstracts of Reviews of Effects (DARE) were examined. The search results revealed a gap in scientific publications concerning objectives like those of this scoping review.

To formulate the research question, the mnemonic combination ‘PCC’ was used, where ‘P’ stands for population, ‘C’ for concept, and ‘C’ for context [25]. The population consisted of studies involving children and adolescents (aged 0 to 19 years) diagnosed with childhood and adolescent cancer, undergoing oncological treatment. The key concept of this scoping review comprised studies detailing coping strategies of parents and/or caregivers within the family dynamic, considering the impact of childhood and adolescent cancer diagnosis on family relationships, parental responsibilities, communication among family members, family routines, as well as problem-solving. The defined context included the home environment, hospital setting, specialized oncology centers, and religious and cultural communities providing social support care. Based on this, the following research question was conceived: “What scientific evidence addresses the effectiveness of dynamic family coping intervention strategies, as well as elements for improving problem-solving skills, for parents and/or caregivers of children and adolescents, aged 0 to 19 years, during and after oncological treatment, considering the impact of childhood and adolescent cancer diagnosis within the family context?”.

### 2.4. Data Collection and Organization

Data collection took place between January and March 2024, from the following databases: PubMed, Cumulative Index to Nursing and Allied Health Literature (CINAHL), Scopus, Web of Science, and Latin American and Caribbean Health Sciences Literature (LILACS).

The search strategy aimed to locate published studies and occurred in two stages. In the first stage, for the search and identification of studies, indexed descriptors in the Health Sciences Descriptors (DeCS) and the Medical Subject Headings (MeSH) were consulted, along with alternative terms suggested by the databases to adapt searches to Portuguese and English. The Boolean operators ‘AND’ and ‘OR’ and truncation were used for the cross-referencing of descriptors and keywords.

In the second stage, a new search using the identified keywords and descriptors was conducted in the databases. For the development of the search strategy, the assistance of an experienced librarian from the University of São Paulo (USP) was enlisted. Modified controlled vocabulary was used for each database. It is worth noting that the search strategy was adapted according to the specificities of each source used; however, combinations between descriptors were preserved; furthermore, time-restriction filters (2014 to 2024) and language-search filters were applied, focusing on Portuguese, English, and Spanish. Additionally, it is highlighted that Table 1 demonstrates the strategies devised with the descriptors used, aided by the Boolean operators ‘AND’ and ‘OR’ to compose the search, as well as quantifying the articles located and selected in each database.

### 2.5. Article Inclusion and Exclusion Criteria

This scoping review included qualitative and quantitative studies, focusing on coping strategies as well as elements for problem-solving, among parents and/or caregivers of children and adolescents, aged 0 to 19 years, undergoing oncological treatment, within the family care context. Qualitative studies of any theoretical and methodological approach were considered, as well as studies published in English, Spanish, or Portuguese, within the timeframe of 2014 to 2024. It is important to emphasize that as the year 2024 has not yet ended, and new studies can still be published in the literature, the search for articles took place up to 31 January 2024. This timeframe is justified due to the intensification of publications and discussions regarding the characterization of coping strategies and problem-solving directed towards childhood and adolescent cancer.

Studies that addressed other types of cancers without focusing on coping and problem-solving, based on literature or theory, were excluded. Additionally, opinion articles, theses, and studies that addressed coping strategies from the perspective of bereaved parents and caregivers were also excluded.

In addition to the criteria mentioned earlier, it is important to highlight the details of the exclusion criteria adopted in this study. Firstly, we excluded studies that did not specifically focus on coping strategies and problem-solving within the specific context of children and adolescents undergoing oncological treatment. This included studies that addressed other types of cancers without a clear emphasis on these specific strategies, as described in relevant studies or pertinent theories. Additionally, we chose to exclude studies that investigated coping strategies from the perspective of bereaved parents and caregivers to maintain thematic cohesion within our scope of review. These criteria were rigorously applied during the study-selection process to ensure the relevance of the included articles and consistency with the objectives and focus of the review. 

### 2.6. Analysis of Results

The characterization of the results began by using the Content Analysis method, which allows for the critical and analytical description of classifying the components of the meaning of messages obtained in articles into different categories, resulting from the grouping of classes of elements that share common characteristics [29]. Discrepancies were resolved through discussion between the two reviewers and, when necessary, by a third reviewer.

All titles and abstracts retrieved in the searches were grouped in the Mendeley^®^ reference-management database for identification and removal of duplicates. For selection, records were exported to the Rayyan web application [30], where duplicates were removed, and two reviewers independently conducted title and abstract screening in a blinded manner. Discrepancies between reviewers were resolved through a consensus meeting with a third reviewer. Subsequently, full-text reading was conducted dynamically. Additionally, a backward search in the references of selected articles was performed to identify possible relevant studies for inclusion in the results.

The classification regarding the level of evidence followed the classification system advocated by JBI [25,26], which comprises five levels of evidence: level 5 (expert opinion), level 4 (descriptive observational studies, such as cross-sectional studies, for example), level 3 (analytical observational studies, such as cohort and case-control studies, for example), level 2 (quasi-experimental studies), and level 1 (experimental studies including systematic reviews and randomized clinical trials). After this step, articles were characterized, and the results related to the research question were synthesized and described and grouped into guiding axes.

Furthermore, aligned with the JBI level of evidence, according to the Oxford Centre for Evidence-based Medicine [30], the lower the number assigned to the study, the better its level of evidence, while studies classified as ‘A’ are considered more relevant, with a higher degree of recommendation. Additionally, it is emphasized that data analysis occurred in tabular form, and data interpretation was descriptive narrative, with tables and statistics elaborated with absolute and relative frequencies. Moreover, the data were compiled into a synthesis table, and each article was randomly assigned an ‘E’ code (study).

Therefore, regarding the data filtering and analysis, it is confirmed that these issues were meticulously conducted as described in the method. After removing duplicates using the Rayyan platform [30], two reviewers independently and blindly screened titles and abstracts, with any discrepancies resolved through consensus in a meeting with a third reviewer. Full-text reading of selected articles was dynamically conducted to ensure accurate inclusion of relevant studies. The classification of evidence levels according to JBI [25,26] and the Oxford Centre for Evidence-based Medicine [30] was rigorously applied, with the synthesis of results organized into thematic axes to provide a clear and comprehensive understanding of the coping strategies and problem-solving approaches adopted by parents and caregivers of children and adolescents during and after oncological treatment.

## 3. Results

### 3.1. Search Results

**Figure 1:**
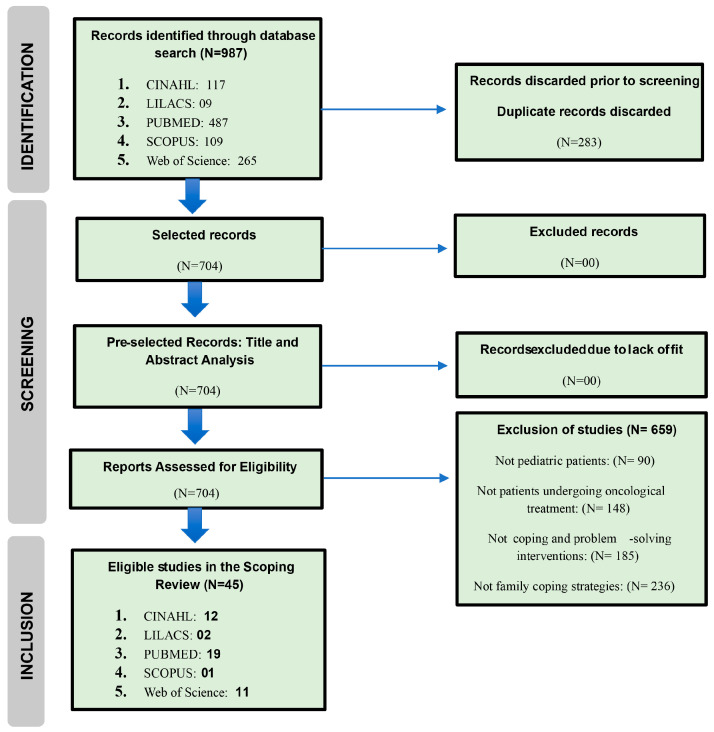
PRISMA flowchart of the study-selection process.

The identification of studies (Figure 1) through databases and records resulted in 987 works. Of this total, 92 studies were fully examined through article reading. Overall, 45 studies were included in the final sample.

Table 2 summarizes the 45 studies with relevant information for the research question, illustrating the data synthesis, coping strategies employed by parents and/or caregivers, and problem-solving skills.

### 3.2. Characteristics of Studies

Regarding the main characteristics observed through the final sampling of eligible studies in this scoping review, it is noteworthy that in terms of the study’s publication location, there was a considerable diversity of countries, totaling 23 nationalities. Additionally, it is highlighted that out of these 23 countries, only the continent of Antarctica was not encompassed in terms of study eligibility. Furthermore, it is inferred that the Asian continent exhibited the highest quantity of studies, comprising 45 studies in the sample (n = 17/37.8%), followed by the American continent (n = 15/33.3%), the European continent (n = 09/20.0%), the African continent (n = 03/6.7%), and the Oceanic continent (n = 01/2.2%). Analyzing each country independently, it is inferred that the United States of America (USA) presented the highest quantity of studies (n = 11/24.4%).

Regarding the methodology employed in each study that comprised the final sample, it is noted that the majority of studies presented level of evidence IV (n = 43/95.6%), indicating qualitative studies. However, it is important to note that although these 43 studies are qualitative, they adopted various methodological approaches, including cross-sectional, descriptive, exploratory, correlational, longitudinal, phenomenological, and mixed-methods studies. It is also noteworthy that two studies presented a level of evidence II (n = 02/4.4%).

Regarding the objectives outlined in each study, within the context of coping strategies and problem-solving, a diversity of purposes was observed, aiming to encompass a wide range of outcomes and, consequently, enhance the understanding of the theme under study. Thus, these objectives encompassed activities of investigation, analysis, identification, description, exploration, examination, evaluation, measurement, and explanation of coping strategies and problem-solving adopted by parents and responsible caregivers during the diagnosis and treatment of children and adolescents with cancer.

**Figure 2:**
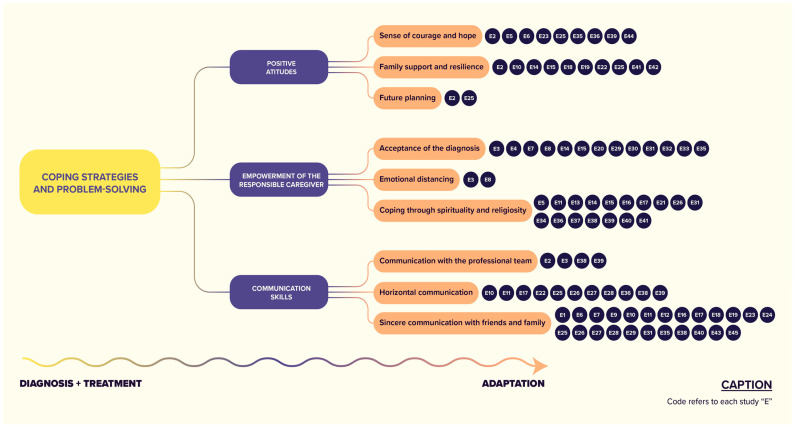
Coping strategies and problem-solving approaches employed by parents and/or caregivers and those responsible for children and adolescents with cancer, from diagnosis to treatment. **Explanatory Note**: The acronyms E1, E2, etc., refer to visual elements of self-identification of study codes, that is, the acronym E1 refers to Study 1, and so on.

Furthermore, based on the findings of each study, it is emphasized that coping strategies were grouped into categories and subcategories as follows: (1) positive attitudes (including a sense of courage and hope, family support to enhance resilience, and future planning), (2) caregiver empowerment (involving acceptance of diagnosis, emotional distancing, and coping through religiosity), and (3) communication skills (encompassing professional communication, horizontal dialogue with healthcare teams, and sincere communication with friends and family) (Figure 2).

### 3.3. Categories and Subcategories of Coping Strategies and Problem-Solving

#### 3.3.1. Positive Attitudes

Positive attitudes in cancer coping refer to the mindset and approaches that patients, caregivers, and family members adopt to deal with the emotional, physical, and psychological challenges associated with the disease. These attitudes involve an optimistic outlook, emotional resilience, acceptance of changes, and a willingness to confront obstacles with courage and hope. Positive attitudes, within the context of coping strategies employed by parents and caregivers of children undergoing cancer treatment, emerge as fundamental components in navigating the multifaceted challenges inherent in the oncological journey. Research demonstrates that these attitudes have a significant impact on psychological well-being, family dynamics, and adaptive skills in the face of adversity. Parents and caregivers with a positive outlook demonstrate greater resilience, fostering an environment of hope, optimism, and proactive problem-solving. Such attitudes not only protect against psychological distress but also empower caregivers to deal with childhood cancer with a sense of efficacy and agency. Additionally, they promote effective communication within the family, strengthening emotional support and facilitating shared decision-making processes. Therefore, cultivating positive attitudes is not just a psychological construct but a dynamic force driving adaptive strategies and family resilience in the face of pediatric cancer diagnosis and treatment [21,31,32,35,36,37,45,53,69,71].

##### Sense of Courage and Hope

Throughout the journey faced by parents and caregivers of children with cancer, a profound sense of courage and hope emerges as a fundamental psychological resource. This challenging context urges them to adopt a stance of resilience, sustained by an unwavering belief in the possibility of overcoming and healing. Courage is manifested in the ability to face difficult moments, from diagnosis to treatment, with determination and bravery, often surpassing their own limits for the well-being of their child or ward. This courage is sustained by hope, a driving force that propels parents and caregivers to persevere even in the face of the uncertainties and challenges inherent in childhood cancer. It is hope that keeps them focused on moments of despair, guiding them through difficulties and providing a beacon of light amidst the darkness. Thus, the sense of courage and hope not only strengthens parents and caregivers but also empowers them to face each stage of the oncological journey with dignity, determination, and a firm commitment to ensuring the best possible care for the child [32,35,40,55,65,66,67,72].

##### Family Support and Resilience

Family support and resilience emerge as essential pillars in coping with and resolving problems during the journey of childhood cancer. The presence of a solid family support network provides an environment of emotional and practical security, where parents and caregivers find emotional support, guidance, and assistance in daily tasks related to the child’s care. This support network not only reduces the impact of emotional stress but also strengthens the ability to cope with the challenges inherent in the child’s oncological condition. Additionally, family resilience, characterized by the ability to adapt and recover from adversity, plays a crucial role in overcoming obstacles. Resilient families can adjust to the changes imposed by the illness, find creative solutions to problems, and maintain a sense of hope and optimism, even in the most difficult circumstances. By cultivating both family support and resilience, parents and caregivers become more equipped to face the challenges of childhood cancer, promoting an environment of loving and sustainable care for the child throughout the treatment and recovery process [32,41,44,45,48,49,52,53,55].

##### Future Planning

In the context of childhood cancer, future planning is essential to provide parents and caregivers with a sense of control and direction. This involves understanding the child’s treatment, considering financial aspects such as medical costs, and cultivating self-care strategies. It also includes establishing social support networks to ensure emotional and practical support. In summary, future planning empowers parents and caregivers to address current challenges while maintaining a hopeful outlook for the future [60].

#### 3.3.2. Empowerment of the Responsible Caregiver

Empowering caregivers of a child with cancer involves providing them with information and resources so they can make informed decisions about treatment. At the same time, it is their responsibility to ensure the best possible care for the child by coordinating family and community support and following medical guidance. This combination of empowerment and responsibility is essential for addressing the challenges of childhood cancer treatment [31,70].

##### Acceptance of the Diagnosis

Acceptance of the diagnosis by parents and caregivers emerges as a vital coping strategy in the context of childhood cancer. Recognizing and accepting the reality of the diagnosis is the first step in dealing with the emotional and practical challenges that accompany the treatment journey. This acceptance allows parents and caregivers to focus their energies on finding solutions and providing the best possible care for the child. Additionally, acceptance of the diagnosis can facilitate the search for emotional and practical support, both within and outside the family, helping parents and caregivers to feel less isolated and more empowered to face the challenges of childhood cancer. In summary, acceptance of the diagnosis is a powerful tool that enables parents and caregivers to confront the reality of the illness and concentrate their efforts on ensuring the well-being of the child [33,44,45,59].

##### Emotional Distancing

Emotional detachment, adopted by some parents and caregivers as a coping mechanism, arises as a strategy for psychological protection in the face of the overwhelming impact of childhood cancer. For some, confronting the emotionally intense reality of diagnosis and treatment can be too overwhelming. Thus, emotional detachment allows for a temporary separation from the painful emotions associated with the situation, helping them to maintain a sense of control and functionality. Although it may seem like detachment, this emotional distancing is often an attempt to preserve one’s own mental health in order to continue caring for the child as effectively as possible. However, it is important to recognize that emotional detachment can also have adverse effects, such as difficulties in emotional communication and in building emotional bonds with the child. Therefore, it is essential for parents and caregivers to have access to emotional support and resources to deal with their own feelings while continuing to support the child during cancer treatment [33,38,43,59,69,71].

##### Coping through Spirituality and Religiosity

Coping through religiosity and spirituality is common among parents and caregivers of children with cancer. Belief in a higher power provides comfort, hope, and purpose, helping to deal with stress and uncertainty. Prayer, meditation, and participation in religious rituals offer a source of inner strength, while the religious community provides support and solidarity. It is important to respect the diverse coping mechanisms of each individual and ensure access to the emotional and spiritual support needed during this challenging journey [35,41,43,44,45,46,47,51,56,64,66,67,68,69].

#### 3.3.3. Communication Skills

Communication emerges as a powerful coping and problem-solving tool for parents and caregivers of children with cancer. Opening frank and open channels of dialogue with the medical team, family, friends, and the child themself is essential for sharing concerns, obtaining information about treatment, and receiving emotional support. Effective communication promotes a deeper understanding of the situation, reducing anxiety and fostering a sense of control over the situation. Additionally, it allows parents and caregivers to express their needs and concerns, facilitating the obtaining of practical and emotional help when necessary. Through communication, parents and caregivers can collaborate with the medical team in decision-making, ensuring that the treatment plan is personalized and tailored to the specific needs of the child [21,31,50,53,68].

##### Communication with the Professional Team

Communication with the multidisciplinary team plays a crucial role in coping with and resolving problems for parents and caregivers of children with cancer. Establishing an open and transparent channel of communication with doctors, nurses, psychologists, and other healthcare professionals allows parents to better understand the diagnosis, treatment plan, and any concerns related to the child’s health. This effective communication also facilitates the exchange of information, enabling parents to express their needs and concerns, while the team provides appropriate guidance and support. Additionally, communication with the multidisciplinary team helps parents feel more empowered and engaged in the child’s care, fostering a sense of partnership in the treatment process. By working together, parents and the multidisciplinary team can develop effective coping strategies and ensure the best possible care for the child during their journey against cancer [32,33,47,52,55,66,68,71].

##### Horizontal Communication

Horizontal communication is an approach in which parents and caregivers establish an equal and collaborative dialogue with healthcare professionals, as opposed to the traditionally hierarchical vertical communication. In this context, parents are encouraged to share their experiences, concerns, and knowledge about the child, while healthcare professionals listen attentively, respecting their perspectives and contributions. This approach fosters an environment of trust, mutual respect, and partnership in decision-making related to the child’s cancer treatment. By enabling a more open and horizontal communication, parents feel more empowered and engaged in the child’s care, while healthcare professionals gain valuable insights that can inform and enhance the treatment plan [34,36,39,40,41,48,56,57].

##### Sincere Communication with Friends and Family

Open and sincere communication with friends and family plays a fundamental role in coping with and resolving problems for parents and caregivers of children with cancer. By openly sharing information about the child’s diagnosis and treatment, parents can receive emotional support, understanding, and solidarity from their loved ones. This frank dialogue also allows friends and family to offer practical help, such as caring for the child’s siblings, preparing meals, or providing transportation to medical appointments. Furthermore, honest communication creates a safe space where parents can express their emotions, fears, and concerns without judgment. This promotes the strengthening of family bonds and the formation of a robust support network, which plays a crucial role in coping with and adapting to the journey of childhood cancer [34,36,37,39,41,42,47,49,53,54,55,56,57,58,61,62,63,65,72,73].

### 3.4. Coping and Problem-Solving

Coping and problem-solving are fundamental processes in adapting to stressful and challenging situations, such as childhood cancer. Coping refers to the cognitive and behavioral strategies used to deal with stress, while problem-solving involves identifying and implementing practical solutions to the challenges faced. There are different types of coping strategies, including active coping, which involves actively seeking solutions, and emotional coping, which focuses on regulating emotions associated with stress. Additionally, social support plays an important role in coping, providing emotional, instrumental, and informational support during difficult times. As for problem-solving, a proactive and solution-focused approach is essential. This may include clearly identifying problems, generating alternative solutions, and implementing effective action plans. Resilience is also a key aspect, allowing individuals to adapt to changes, learn from challenging experiences, and continue moving forward, even in the face of adversity [74,75,76].

## 4. Discussion

The diagnosis of childhood cancer is a highly challenging moment for families, demanding adaptive strategies that not only support the sick child but also strengthen the family unit as a whole. The body of evidence from the scoping review, which encompassed 45 studies, revealed a wide range of strategies employed by parents and caregivers, highlighting the crucial importance of family support, religiosity, effective communication, and caregiver empowerment to facilitate positive adaptation during treatment [40,62].

Consistent with previous studies, the presence of positive attitudes such as courage and hope emerge as a fundamental pillar in coping with childhood cancer. Smith et al. (2024) and Luo et al. (2021) emphasize that these attitudes not only strengthen caregivers’ emotional resilience but also directly influence the children’s psychological well-being, creating a positive support environment around them [72,77].

The family support network proves to be a determining factor for better emotional and adaptive outcomes during treatment. Schoors et al. (2019) and Melguizo-Garín et al. (2023) indicate that families with strong social support tend to more effectively handle the stress and uncertainty associated with childhood cancer, highlighting the importance of policies and practices that strengthen this support network. Additionally, Nukpezah et al. (2021) found that in Ghana, parents often turn to community support and religion, underscoring the universality of the importance of social and spiritual support [52,53,54,55,56,57,58,59,60,61,62,63,64,65,66,67,68,69,70,71,72,73,74,75,76,77,78,79].

Beyond family support, strategies such as caregiver empowerment have been identified as essential for psychological adaptation and practical support provided to the sick child. Lin et al. (2019) and Mardhyah et al. (2022) discuss how the acceptance of the diagnosis, the use of emotional distancing strategies, and engagement in religious practices help caregivers face the emotional and practical challenges associated with childhood cancer. This finding is reinforced by the study of Deribe et al. (2023), which identified that acceptance of the child’s condition and effective communication with healthcare professionals are essential coping strategies among parents in Ethiopia [68,80,81].

Communication skills, both with the healthcare team and with friends and family, emerge as a fundamental competence to facilitate mutual understanding and continuous support. Smith et al. (2024) and Lin et al. (2019) stress that open and horizontal communication not only promotes personalized care but also strengthens the emotional support needed to face the ups and downs of pediatric oncology treatment [72,80].

Spirituality also plays a crucial role in coping, providing hope and emotional support to parents during their child’s treatment, as found by Chong et al. (2023) among Malay Muslim caregivers of children with acute lymphoblastic leukemia. This spiritual dimension, along with religiosity, is a significant coping strategy that can provide a solid emotional and practical foundation during pediatric oncology treatment [67].

The findings significantly contribute to the field by offering an in-depth and systematic analysis of the coping strategies utilized by parents and caregivers. By categorizing and integrating these strategies, we provide a holistic view of adaptive practices that can be implemented to improve the emotional and practical support offered to these families. Furthermore, the importance of targeted interventions that strengthen positive attitudes, empower caregivers, and promote effective communication is highlighted as essential components in comprehensive care during pediatric oncology treatment [39,40,41,42,46,49].

The three broad themes synthesized from the studies that comprised the final sample of this scoping review highlighted the significant impact of oncological disease on the lives of parents and/or caregivers of children and adolescents, as well as on family dynamics, greatly affecting these caregivers’ ability to cope with situations from diagnosis to treatment continuity. Thus, it is noted that the findings were similar to reviews that focused on pediatric oncological themes [31,32,33,34,35,36,37,38] or included cancer as part of a variety of chronic diseases, as well as life-threatening conditions [39,40,41,42,46,49].

In terms of impact on family functioning, studies (E24 to E32) also reported that parents and caregivers experienced significant disruption in routine and family relationships [54,55,56,57,58,59,60,61,62]. Regarding coping and psychological well-being, qualitative research, evidenced by studies (E35, E43, E45), indicated that parents and caregivers of children and adolescents affected by neoplasms experienced intense feelings with unmet needs, leading to emotional struggles and the adoption of inadequate coping mechanisms (acting out at home) [65,72,73].

In terms of caregivers, much of their social well-being is related to the coping mechanisms they employ, such as the following: (1) positive attitudes, including a sense of courage and hope, family support to enhance resilience, and future planning; (2) caregiver empowerment, involving acceptance of the diagnosis, emotional distancing, and coping through religiosity; and (3) communication skills, encompassing professional communication, horizontal dialogue with healthcare teams, and sincere communication with friends and family [40,43,72]. These caregivers often report difficulties within the family environment, frequently resorting to emotional distancing as a means to strengthen their ability to continue providing care [59]. Additionally, they face overwhelming uncertainties related to the child’s health condition and experience anxiety within the context of family caregiving [38,70]. These findings underscore the pressing need for further research in this area to identify the causes of caregivers’ distress and develop effective support strategies.

Furthermore, it is noteworthy that the burden of care in cancer-related conditions can extend over many years, placing immense strain on the physical, financial, and emotional resources of families and may involve the use of technology (such as feeding tubes and ventilators) [69,72]. Future research should encompass parents and caregivers responsible for children and adolescents with cancer from a variety of life-threatening conditions, particularly those with diagnoses related to pediatric oncology, and with sample sizes large enough to allow for sub-analyses based on different aspects of the child’s illness [51,61,64].

The utilization of theoretical frameworks to guide family or illness-related research assists researchers in contextualizing observed problems. Thus, it is emphasized that studies (E22, E32, E36) strengthen their methodology, results, and discussions by grounding themselves in theoretical frameworks, as they enrich the interpretation of findings and influence clinical practice and future research [52,62,66]. Additionally, studies (E38, E40) have also noted that the absence of a theoretical framework diminishes the quality of research on the proposed topic [21,68]. In this regard, the significance of family relationships in shaping the experience of these parents and caregivers provides a family-focused perspective on familial dynamics [67]. Furthermore, the adoption of a specific model should be a priority to advance knowledge in this field [16,63].

### 4.1. Study Limitations

The present scoping review delineates some noteworthy limitations. Firstly, it is imperative to acknowledge that while the search strategy was meticulously devised to encompass all relevant studies, it is plausible that some may have inadvertently been omitted. For instance, studies involving parents and/or caregivers of children with cancer, focusing on broader aspects of family well-being, may have eluded detection during the established review process.

### 4.2. Implications for Nursing Practice

The evidence presented in this study represents a significant contribution to nursing in formulating a care protocol aimed at family caregivers. The ability to provide care, still an emerging theme in studies, provides an understanding of individuals’ conditions to take on caregiving responsibilities. From this perspective, it is possible to provide essential support for nursing practice in developing attributes of knowledge, courage, and patience. Implementing support and guidance actions to strengthen family caregivers in the context of home care can reduce burden and stress, and promote the development of effective coping strategies, thus mitigating the negative impact of these adversities on caregiving ability.

In this context, nurses play a crucial role in identifying the emerging needs of this population. Through collaboration with other healthcare professionals and different levels of care, they can develop strategies to support caregivers, including health education actions and psychological support, with the aim of ensuring adequate care and promoting quality of life for caregivers facing the challenge of home care.

Furthermore, the exploration of coping strategies used by parents and caregivers should be conducted on an individualized basis. Coping support can be facilitated by helping children and their families identify distressing symptoms and exploring strategies that may be helpful in mitigating negative impacts on quality of life.

### 4.3. Future Research Direction

Families play a crucial role in the development, overall well-being, and coping abilities when there is a child with cancer in the family. Future research could consider exploring the roles of extended family members (such as grandparents), neighbors, and the broader community (including youth clubs and religious or cultural communities) in providing additional support to parents and caregivers. These extended support networks may facilitate better support for these caregivers. Additionally, future work may consider the influence of cultural beliefs or traditions on family adaptation. Researchers should consider designing longitudinal studies on the psychosocial outcomes or coping strategies of parents and caregivers, as well as engaging in more in-depth qualitative approaches. These results may help parents, clinicians, and researchers recognize patterns of change in family dynamics over time and serve as a basis for the development of interventions specific to the unique needs of these caregivers. Furthermore, it is noteworthy that longitudinal studies can also clarify the resilience of parents and caregivers and how support can be directed to strengthen their coping skills.

## 5. Conclusions

Initially, it is imperative to highlight that coping strategies and problem-solving play a significant role in the process of acceptance and reframing of ideas and feelings related to childhood and adolescent cancer by families. In this context, it becomes crucial that nursing interventions and those of related professionals, both during diagnosis and after treatment, be geared towards empowering families to adopt a range of resources. These include seeking information and understanding about pediatric neoplasms and their future implications, fostering effective intrafamily communication, providing support in delivering specific care to this population, as well as adopting planning and preparation strategies for the inevitable changes that will occur in the family dynamic. Additionally, it is crucial to establish links with support groups composed of families of children with cancer.

As time progresses, such resources bring about changes in family functioning patterns and their interactions with the external environment. During this period, coordinated professional practices become essential to support families in making relevant decisions regarding the child’s development and growth, as well as to assist them in managing the tensions that arise along the way. These practices include encouraging communication and decision-sharing within the family, recognizing and promoting family strengths and beliefs, as well as valuing the unique qualities inherent in a child with cancer.

It is imperative to emphasize, however, that the mere existence of coping strategies and problem-solving does not guarantee adequate adaptation. However, identifying such strategies allows for the presentation of resources that can make the process more manageable for the family unit. In this context, nurses and other healthcare professionals can utilize the findings outlined here as indicators to encourage families to deal more effectively with the situations involving the diagnosis and treatment of cancer in children and adolescents.

Based on the analysis addressed in this scoping review, we can conclude that the care of children and adolescents during and after oncological treatment poses significant challenges for parents and caregivers. The reviewed studies highlighted the substantial impact of the disease on the lives of these caregivers, demonstrating disruptions in family routines and intense emotional relationships. Coping strategies, such as positive attitudes, caregiver empowerment, and communication skills, were identified as crucial for dealing with the demands of care. However, the lack of consistency in the measures used and the absence of control for variables such as the patients’ age and sex represent significant methodological challenges. Furthermore, the lack of information on the time from diagnosis to the start of oncological treatment complicates the understanding of the disease’s impact over time. To advance in this area, longitudinal studies are needed that consider these aspects and employ theoretical frameworks to guide research. By doing so, we can develop more effective interventions to support parents and caregivers throughout the course of their children’s oncological treatment.

## Figures and Tables

**Table 1:** Search strategies for articles in databases, 2014–2024.

Database	Search Strategy
PubMed (n = 487)	(“Coping” OR “Coping Strategy” OR “Coping” OR “Problem solving” OR “Coping Skills” OR “Effectiveness”) AND (“Family” OR “Caregivers” OR “Family dynamics” OR “Family communication” OR “Family relationship”) AND (“Adaptation” OR “Psychological” OR “Adaptation strategy” OR “Adaptive Behavior”) AND (Child OR Children OR Childhood OR Pediatric OR adolescent* OR adolescent* OR childhood cancer survivors) AND (oncology OR neoplasms* OR tumor* OR Cancer*) AND (“Family environment” OR “Home environment” OR “Hospital environment “ OR “Specialized centers” OR “Religious communities” OR “Cultural communities” OR “Social support”)
CINAHL (n = 117)	(“Coping” OR “Coping Strategy” OR “Coping” OR “Problem solving” OR “Coping Skills” OR “Effectiveness”) AND (“Family” OR “Caregivers” OR “Family dynamics” OR “Family communication” OR “Family relationship”) AND (“Adaptation” OR “Psychological” OR “Adaptation strategy” OR “Adaptive Behavior”) AND (Child OR Children OR Childhood OR Pediatric OR adolescent* OR adolescent* OR childhood cancer survivors) AND (oncology OR neoplasms* OR tumor* OR Cancer*) AND (“Family environment” OR “Home environment” OR “Hospital environment “ OR “Specialized centers” OR “Religious communities” OR “Cultural communities” OR “Social support”)
SCOPUS (n = 109)	(“Coping” OR “Coping Strategy” OR “Coping” OR “Problem solving” OR “Coping Skills” OR “Effectiveness”) AND (“Family” OR “Caregivers” OR “Family dynamics” OR “Family communication” OR “Family relationship”) AND (“Adaptation” OR “Psychological” OR “Adaptation strategy” OR “Adaptive Behavior”) AND (Child OR Children OR Childhood OR Pediatric OR adolescent* OR adolescent* OR childhood cancer survivors) AND (oncology OR neoplasms* OR tumor* OR Cancer*) AND (“Family environment” OR “Home environment” OR “Hospital environment “ OR “Specialized centers” OR “Religious communities” OR “Cultural communities” OR “Social support”)
Web of Science (n = 265)	(“Coping” OR “Coping Strategy” OR “Coping” OR “Problem solving” OR “Coping Skills” OR “Effectiveness”) AND (“Family” OR “Caregivers” OR “Family dynamics” OR “Family communication” OR “Family relationship”) AND (“Adaptation” OR “Psychological” OR “Adaptation strategy” OR “Adaptive Behavior”) AND (Child OR Children OR Childhood OR Pediatric OR adolescent* OR adolescent* OR childhood cancer survivors) AND (oncology OR neoplasms* OR tumor* OR Cancer*) AND (“Family environment” OR “Home environment” OR “Hospital environment “ OR “Specialized centers” OR “Religious communities” OR “Cultural communities” OR “Social support”)
LILACS (n = 09)	(Criança OR Crianças OR Infância OR Pediátrico OR adolescente* OR adolescente* OR sobreviventes de câncer infantil OR Habilidades de enfrentamento OR Efetividade) AND (oncologia OR neoplasias* OR tumor* OR Câncer*))) AND (“Coping” OR “Estratégia de Coping” OR “Enfretamento” OR “Resolução de problemas” OR “Família” OR “Cuidadores” OR “Dinâmica familiar” OR “Diagnóstico” OR “Comunicação familiar” OR “Relação familiar”)) AND (“Ambiente familiar” OR “Ambiente doméstico” OR “Ambiente Hospitalar” OR “Centros especializados” OR “Comunidades religiosas” OR “Comunidades culturais” OR “Suporte social”)

**Explanatory Note:** The asterisk (*) was used as a truncation operator in the search strategy to include all possible variations of the keywords. This operator allows for the retrieval of terms with different suffixes or morphological variations, thus broadening the search scope. For example, the term “neoplasms” includes both “neoplasms” and “neoplasm”; “adolescent*” retrieves both “adolescent” and “adolescents”. The use of the asterisk ensures that the search is comprehensive and includes all possible forms of the relevant words for the study.

**Table 2:** Synopsis of studies included in the scoping review.

Code/Authorship/Year of Publication/Country of Study Publication/Journal	Methodological Design/Sample Size/Level of Evidence	Studies Objectives	Key Syntheses (Coping Strategies, Coping Skills, and Problem-Solving)
(E1)/Woźniak et al./2014/Poland/Menopause Review [31]	Qualitative Study/n = 10 families were included/Level of Evidence (IV)	Describing the nature of coping skills developed by the family in caring for the cancer patient.	(1) Positive attitudes; (2) empowerment aimed at caring for the child with cancer; and (3) necessary communication skills related to health, to clarify the child’s condition.
(E2)/Pei-Fan et al./2015/Taiwan/JBI Evidence Synthesis [32]	Qualitative study/n = 08 qualitative articles were included in the review/Level of Evidence (IV)	Understanding the experiences of family members during the year following the diagnosis of cancer in a child or adolescent in their family.	(1) Familial loss and the turmoil surrounding the cancer diagnosis; (2) a sense of courage and hope of mutual responsibility inspired by changes in circumstances; (3) increased family support enhancing the resilience of family members; (4) healthcare professional–patient communication; and (5) a positive attitude towards the illness and future planning.
(E3)/Popp et al./2015/United States/Journal of Pediatric Oncology Nursing [33]	Qualitative study, through semi-structured interviews/n = 50 parents were included (n = 46 mothers and n = 04 fathers)/Level of Evidence (IV)	(1) Evaluate the experience of parents with a child diagnosed with cancer; (2) analyze parents’ hope, family functioning, and caregiving perceptions that distinguish parents who have adapted to the diagnosis versus those who have not.	(1) Acceptance of the diagnosis; (2) conflicting thoughts about the child’s diagnosis; (3) emotional distancing of parents from their children as a resilience behavior; (4) adaptation time to the diagnosis and treatment; and (5) effective communication with healthcare professionals.
(E4)/Nóia et al./2015/Brazil/Investigación y Educación en Enfermería—Research and Education in Nursing [34]	Qualitative study, with descriptive-exploratory design/n = 10 families were included/Level of Evidence (IV)	Analyzing how family members cope with the hospitalization scenario due to the diagnosis of childhood cancer.	(1) Family coping in the face of diagnosis; and (2) family coping in the face of hospitalization.
(E5)/Van der Geest et al./2015/Netherlands/Journal of Palliative Medicine [35]	Qualitative study/n = 89 parents were included/Level of Evidence (IV)	(1) Explore the role of faith and hope as sources of coping; (2) analyze long-term parental adjustment as an indicator.	(1) Coping through faith; (2) coping through hope; and (3) feelings of possibility of cure.
(E6)/He et al./2016/China/Cancer Nursing [36]	Qualitative study, with descriptive correlational approach/n = 95 parents were included/Level of Evidence (IV)	(1) Measure uncertainty, coping strategies, and growth through uncertainty (GTU); (2) explore the relationships among parents of children with acute leukemia in China; (3) explore factors related to parental uncertainty regarding coping skills.	(1) Reordering and reorganization of family priorities; (2) positive strategies (optimism) for coping with stress and unpredictability surrounding treatment.
(E7)/Lakkis et al./2016/Lebanon/Psycho-Oncology [37]	Qualitative cross-sectional study/n = 114 parents were included (n = 85 mothers and n = 29 fathers)/Level of Evidence (IV)	(1) Determine the prevalence of psychological distress (PD) among parents of Lebanese children with cancer; (2) investigate associated stressors and coping strategies used by parents.	(1) Financial adjustment of families and parental job stability as a form of coping; (2) optimistic outlook regarding treatment; and (3) family integration.
(E8)/Penner et al./2016/United States/Clinical Psychological Science [38]	Longitudinal study/n = 99 parents were included/Level of Evidence (IV)	(1) Examine variability: caregivers’ trends toward self-distancing when reflecting on their feelings about their children’s treatments; (2) measure caregivers’ anxiety levels at the study’s outset, anticipatory anxiety during treatment procedures, and caregivers’ psychological distress.	(1) Self-distancing as a coping strategy (self-distancing protected caregivers of children with cancer with more pronounced anxiety traits from short- and long-term distress, without promoting treatment avoidance).
(E9)/Cox/2016/Australia/European Journal of Cancer Care [39]	Qualitative study through semi-structured interviews/n = 38 parents included/Level of Evidence (IV)	Examine parents’ experiences and coping strategies during their child’s cancer diagnosis.	(1) Family coping upon diagnosis; and (2) family coping during hospitalization.
(E10)/Hendricks-Ferguson et al./2017/United States/Journal of Pediatric Hematology/Oncology Nursing [40]	Pilot study of multicenter, prospective, longitudinal single-group design/n = 11 families were included/Level of Evidence (II)	(1) To determine the feasibility, acceptability, and responses related to a communication intervention (coping) titled “Communication Plan: From Diagnosis to End of Life” for parents of children with brain tumors.	(1) Coping with decisions; (2) reframing hope; (3) reducing uncertainties related to treatment; and (4) effective horizontal communication with the multidisciplinary team.
(E11)/Doumit et al./2017/Lebanon/Journal of Psychosocial Oncology [41]	Qualitative study, through semi-structured interviews/n = 11 families were included/Level of Evidence (IV)	To gain a deep understanding of the factors that facilitate and hinder coping methods for Lebanese parents with a child with cancer.	(1) Social and family support; (2) strong religious beliefs; (3) effective horizontal communication with the multidisciplinary team; and (4) sincere and open communication with family members.
(E12)/Wiener et al./2017/United States/Psychooncology [42]	Qualitative, cross-sectional, multicenter study, through questionnaire application and semi-structured interviews/n = 192 parents included/Level of Evidence (IV)	(1) Explore how having a child with cancer affects the quality of family relationships; (2) identify factors that help couples remain emotionally engaged during childhood cancer treatment; (3) evaluate parents’ interest in counseling intervention.	(1) Coping through family counseling (family therapy); (2) emotional connection; and (3) family adjustment.
(E13)/Abdoljabbari et al./2018/Somalia/Asian Pacific Journal of Cancer Prevention [43]	Qualitative study through semi-structured interviews/n = 15 parents were included/Level of Evidence (IV)	Evaluate the spiritual state as a coping strategy of parents of children with cancer.	(1) Spiritual coping; (2) spiritual avoidance; and (3) spiritual growth during the treatment process.
(E14)/Chivukula et al./2018/India/Indian Journal of Palliative Care [44]	Qualitative study, through semi-structured interviews/n = 100 parents included (n = 50 mothers and n = 50 fathers)/Level of Evidence (IV)	(1) Determine whether mothers and fathers of children suffering from acute lymphoblastic leukemia (ALL) differ in coping and spirituality; (2) determine if there is a relationship between caregiver burden dimensions, coping, and spirituality among caregivers of children with ALL; (3) determine if coping and spirituality predict caregiver burden.	(1) Coping through religion; (2) acceptance of the child’s health condition; (3) use of instrumental support; (4) use of emotional support; and (5) coping through ecological awareness.
(E15)/Cutillo et al./2018/Great Britain/Journal of Neurosurgery: Pediatrics [45]	Qualitative study, through semi-structured interviews/n = 40 parents included/Level of Evidence (IV)	To determine which coping strategies caregivers employ to deal with the stress of a child recently undergoing surgical treatment for a newly diagnosed brain tumor.	(1) Active coping; (2) acceptance coping; (3) emotion-focused coping; (4) spiritual coping; (5) social support; and (6) post-traumatic growth.
(E16)/Nikfarid et al./2018/Iran/Journal of Pediatric Hematology/Oncology Nursing [46]	Qualitative study, using purposive sampling/n = 08 mothers were included/Level of Evidence (IV)	Explain the dimensions of religious coping in mothers of children with cancer in Iran.	(1) Coping through religion; (2) emotion regulation; (3) reframing uncertainties about treatment; and (4) establishing a new family dynamic.
(E17)/Doumit et al./2019/Lebanon/European Journal of Oncology Nursing [47]	Qualitative study, through semi-structured interviews/n = 11 families were included/Level of Evidence (IV)	To understand the meaning of spirituality as a coping mechanism for parents of cancer patients in Lebanon.	(1) Coping through spirituality; (2) connection and closeness with other parents of children with cancer; (3) effective and transparent communication with the multidisciplinary team.
(E18)/Clever et al./2019/Germany/Family Process [48]	Qualitative Study, Cross-Sectional/n = 108 Parents Included/Level of Evidence (IV)	(1) Investigate individual and dyadic coping strategies of mothers and fathers of children with hematologic cancer; (2) analyze how these strategies relate to Fear of Progression (FoP).	(1) Coping through family interaction; and (2) maintenance of psychological support.
(E19)/Lyu et al./2019/China/Cancer Nursing [49]	Qualitative study, with mixed methods/n = 212 families were included/Level of Evidence (IV)	Assess the perceived family impact and coping during the child’s hospitalization for acute lymphoblastic leukemia (ALL), treatment; (2) identify potential predictors of perceived family impact.	(1) Coping through psychological and therapeutic support strategies; (2) change in family dynamics; (3) social support from friends and family.
(E20)/Paula et al./2019/Brazil/Revista Cuidarte [50]	Descriptive, cross-sectional study with a qualitative approach/n = 27 caregivers were included/Level of Evidence (IV)	Understand how families cope with the diagnosis of childhood cancer.	(1) Optimistic approach to diagnosis and treatment; and (2) knowledge as a form of relief.
(E21)/Díaz-Morales et al./2019/Mexico/Revista Cuidarte [51]	Correlational descriptive study/n = 31 family members were included/Level of Evidence (IV)	Describe and analyze the relationship between symptoms of pain, nausea, and vomiting in children with cancer and describe family care strategies when faced with these symptoms.	(1) Coping through religious belief; and (2) music therapy.
(E22)/Schoors et al./2019/Belgium/Frontiers of Psychology [52]	Qualitative, cross-sectional study/n = 123 parents were included (n = 70 mothers and n = 53 fathers)/Level of Evidence (IV)	(1) Explore the role of protective factors at the individual (parental psychological flexibility), intrafamily (dyadic coping), and contextual level (support network) in explaining family adjustment as a strategy perceived by parents of children with leukemia or non-Hodgkin’s lymphoma; (2) analyze whether protective factors could be predictive elements for the family later.	(1) Coping through psychological support; (2) network support; and (3) direction of interventions by the multidisciplinary team.
(E23)/Lyu et al./2019/China/Journal of Pediatric Nursing [53]	Descriptive qualitative study/n = 24 parents were included/Level of Evidence (IV)	Explore how Chinese families cope with children being hospitalized for cancer treatment.	(1) Coping through family interaction (emotional closeness); (2) optimistic thoughts; (3) external support; and (4) truthful information about your child’s health condition.
(E24)/Salvador et al./2019/Portugal/Psycho-Oncology [54]	Qualitative, cross-sectional study/n = 205 parents were included/Level of Evidence (IV)	Examine the contribution of individuals (positive reappraisal) and family factors (parental satisfaction, couple relationship, quality of life, and difficulties in family life) to the psychological well-being (PWB) of parents of children/adolescents diagnosed with cancer.	(1) Coping through the positive reappraisal strategy; and (2) change in family dynamics.
(E25)/Liu et al./2020/China/Journal of Pediatric Hematology/Oncology Nursing [55]	Qualitative study, using in-depth face-to-face interviews/n = 10 parents were included/Level of Evidence (IV)	To describe parental experiences of having a child with acute lymphoblastic leukemia (ALL) in China.	(1) Coping through parental resilience; (2) coping through perceived hope; (3) support services to strengthen families’ specific protective factors (i.e., family/community support) and positive coping; (4) effective communication with the healthcare team (through educational materials); and (5) coping through ongoing personalized interventions.
(E26)/Padeniya et al./2020/India/Acta Oncologica [56]	Qualitative, cross-sectional study/n = 200 mothers were included/Level of Evidence (IV)	To evaluate maternal coping and strategies in response to their children with cancer in Sri Lanka.	(1) Coping through family integration; (2) cooperation between family members; (3) optimistic definition of your child’s healing; (4) horizontal communication with the multidisciplinary team; and (5) coping through religiosity.
(E27)/Tan et al./2020/Singapore/Clinical Nursing Research [57]	Qualitative study, using semi-structured interviews/n = 10 mothers were included/Level of Evidence (IV)	Explore caregiving stress, coping strategies, and support needs of mothers caring for children/adolescents with cancer during the active treatment phase.	(1) Coping through the support network (friends, family and healthcare team); and (2) coping through educational booklets on cancer treatment protocols.
(E28)/Omari et al./2021/Oman/Cancer Nursing [58]	Qualitative study/n = 10 mothers were included/Level of Evidence (IV)	Explore the lived experiences of Omani mothers caring for children with leukemia, using an interpretive phenomenological analysis design.	(1) Coping through viable support systems (other mothers, family members, and professional staff).
(E29)/López et al./2021/Spanish/European Journal of Cancer Care [59]	Qualitative interpretative phenomenological analysis study (IPA)/n = 10 caregivers were included/Level of Evidence (IV)	Explore the mediating role of emotional avoidance and acceptance in parents’ emotional adjustment throughout illness.	(1) Coping through acceptance of the oncological diagnosis; (2) parental emotional self-avoidance; and (3) change in family dynamics.
(E30)/Koumarianou et al./2021/Greece/Palliative and Supportive Care [60]	Qualitative study, through an integrative literature review/n = 17 qualitative studies were included/Level of Evidence (IV)	Evaluate the evidence on psychosocial interventions aimed at families during their children’s active lives in cancer treatment and make recommendations for the direction of future research.	(1) Coping through cognitive-behavioral therapy strategies; and (2) training in problem-solving skills aimed at maternal distress.
(E31)/Yeung et al./2021/China/International Journal of Environmental Research and Public Health [61]	Qualitative study/n = 15 parents were included/Level of Evidence (IV)	Describe stressors and psychosocial effects on adaptation experienced by Chinese parents of children with cancer or hematological disease disturbances in Hong Kong during the transition and survival phases.	(1) Coping by focusing on the problem; (2) seek information to alleviate your concern; (3) talk to other parents who deal with the same problem; (4) seek social support from NGOs; (5) positive mindset; (6) signifying illness through faith; (7) coping through work; and (8) coping through meditation.
(E32)/Basile et al./2021/United States/Journal of Pediatric Psychology [62]	Pilot study without randomization/n = 238 caregivers were included/Level of Evidence (II)	To examine the roles of constructive and dysfunctional problem-solving strategies in the relationships between illness uncertainty and adjustment outcomes (i.e., anxious, depressive, and posttraumatic stress symptoms) in caregivers of children newly diagnosed with cancer.	(1) Coping through dysfunctional problem-solving strategies; and (2) adaptation to your child’s oncological diagnosis.
(E33)/Miller et al./2022/United States/Cancer Medicine [63]	Qualitative study/n = 36 caregivers were included/Level of Evidence (IV)	Understand how caregivers of children with brain tumors use social media as a coping strategy.	(1) Coping using social media such as Facebook, sharing information about your children’s diagnosis and ways of positive coping with other families with the same health condition.
(E34)/Farinha et al./2022/Brazil/Revista Bioética [64]	Qualitative, descriptive and cross-sectional study/n = 30 informal caregivers were included/Level of Evidence (IV)	To identify the use of religious/spiritual coping in informal caregivers of children with acute lymphocytic leukemia through application of the brief religious/spiritual coping scale.	(1) Coping through religiosity and spirituality.
(E35)/Wang et al./2022/China/Cancer Nursing [65]	Qualitative study, using semi-structured interviews/n = 32 caregivers were included/Level of Evidence (IV)	To study the psychological adaptation process of parents caring for pediatric patients with leukemia.	(1) Coping through hope; (2) family integration; and (3) psychological adaptation.
(E36)/Eche et al./2022/United States/Cancer Nursing [66]	Qualitative study, through systematic review/n = 17 studies were included/Level of Evidence (IV)	To comprehensively describe the experiences of hope in parents of children with cancer.	(1) Coping through hope; (2) coping through religiosity and spirituality; and (3) coping through adequate communication with the multidisciplinary team.
(E37)/Koutelekos et al./2023/Greece/Advances in Experimental Medicine and Biology [16]	Qualitative study/n = 85 parents were included (n = 65 mothers and n = 20 fathers)/Level of Evidence (IV)	Explore the coping strategies used by Greek parents who have children with cancer.	(1) Coping through religiosity.
(E38)/Mensah et al./2023/Ghana/BMC Psychology [21]	Qualitative, descriptive, phenomenological study, using semi-structured interviews/n = 20 caregivers were included/Level of Evidence (IV)	Explore the tensions, resources, and coping strategies of families and caregivers of children and adolescents diagnosed with cancer in Ghana.	(1) Coping through religiosity; (2) self-motivation; (3) family cohesion; and (4) community support.
(E39)/Chong et al./2023/Malaysia/Asia Pacific Journal of Public Health [67]	Qualitative, descriptive study/n = 13 parents were included/Level of Evidence (IV)	Exploring spirituality among Malaysian Muslim caregivers of children with acute lymphoblastic leukemia.	(1) Coping through hope; and (2) coping through spirituality.
(E40)/Deribe et al./2023/Ethiopia/BMJ Open [68]	Qualitative, phenomenological study/n = 21 parents were included (n = 15 mothers and n = 06 fathers)/Level of Evidence (IV)	Explore sources of stress, conditions that help reduce stress levels, and coping strategies among parents of children with cancer.	(1) Addressing the child’s health condition through counseling by the multidisciplinary team; (2) social support; (3) acceptance of the child’s condition; (4) spirituality; and (5) communication with healthcare providers.
(E41)/Ochoa-Dominguez et al./2023/United States/International Journal of Environmental Research and Public Health [69]	Qualitative study, using semi-structured interviews/n = 15 parents were included/Level of Evidence (IV)	Describe the psychological health of Hispanic parents and explore their coping strategies.	(1) Problem-focused coping (self-efficacy, behavioral change and social support); (2) emotion-focused coping (religious practices and positive reframing); and (3) avoidant coping (denial and self-distraction).
(E42)/Phiri et al./2023/China/Psycho-Oncology [70]	Qualitative study, through systematic review with meta-analysis/n = 14 studies were included/Level of Evidence (IV)	Assess the evidence on the effectiveness of psychoeducational interventions (PEIs) in reducing anxiety and depressive symptoms, improving health-related quality of life (HRQoL) and coping skills in caregivers of children with cancer.	(1) Coping through psychoeducational interventions.
(E43)/Bates et al./2023/United States/Journal of Pediatric Nursing [71]	Qualitative, cross-sectional study/n = 44 parents were included/Level of Evidence (IV)	Gather caregivers’ perspectives on barriers and facilitators to adaptive family functioning during the early stages of cancer treatment, with a focus on family rules and routines.	(1) Coping through the support network; and (2) coping through routines and rules in the family nucleus.
(E44)/Smith et al./2024/United States/Journal of Psychosocial Oncology [72]	Qualitative study, using semi-structured interviews/n = 183 parents were included/Level of Evidence (IV)	Identify links between caregiver hope, caregiver coping behaviors, and caregiver training versus rejection of emotional socialization (ES) beliefs in a pediatric cancer sample.	(1) Coping through hope; and (2) emotional coaching.
(E45)/Bates et al./2024/United States/Journal of Pediatric Psychology [73]	Cross-sectional qualitative study/n = 86 parents were included/Level of Evidence (IV)	Quantify family involvement during pediatric cancer treatment and associations with children’s emotional and behavioral health.	(1) Coping through routines and rules in the family nucleus.

## Data Availability

No new data were created or analyzed in this study. Data sharing is not applicable to this article.

## Guidelines and Standards Statement

This manuscript was drafted following the Preferred Reporting Items for Systematic Reviews and Meta-Analyses (PRISMA) Statement.
